# The Economic Crisis and Acute Myocardial Infarction: New Evidence Using Hospital-Level Data

**DOI:** 10.1371/journal.pone.0142810

**Published:** 2015-11-17

**Authors:** Aleksandra Torbica, Aldo Pietro Maggioni, Simone Ghislandi

**Affiliations:** 1 Department of Policy Analysis and Public Management - Centre for Research on Health and Social Care Management (CERGAS), Bocconi University, Milan, Italy; 2 Italian Association of Hospital Cardiologists (ANMCO) Research Center, Florence, Italy; 3 Dondena Centre for Research on Social Dynamics and Public Policy, Bocconi University, Milan, Italy; University of Bologna, ITALY

## Abstract

**Objective:**

This research sought to assess whether and to what extent the ongoing economic crisis in Italy impacted hospitalizations, in-hospital mortality and expenditures associated with acute myocardial infarction (AMI).

**Methods:**

The data were obtained from the hospital discharge database of the Italian Health Ministry and aggregated at the hospital level. Each hospital (n = 549) was observed for 4 years and was geographically located within a “Sistema Locale del Lavoro” (SLL, i.e., clusters of neighboring towns with a common economic structure). For each SLL, the intensity of the crisis was determined, defined as the 2012–2008 increase in the area-specific unemployment rate. A difference-in-differences (DiD) approach was employed to compare the increases in AMI-related outcomes across different quintiles of crisis intensity.

**Results:**

Hospitals located in areas with the highest intensity of crisis (in the fifth quintile) had an increase of approximately 30 AMI cases annually (approximately 13%) compared with hospitals in area with lower crisis intensities (p<0.001). A significant increase in total hospital days was observed (13%, p<0.001) in addition to in-hospital mortality (17%, p<0.001). As a consequence, an increase of around €350.000 was incurred in annual hospital expenditures for AMI (approximately 36%, p<0.001).

**Conclusions:**

More attention should be given to the increase in health needs associated with the financial crisis. Policies aimed to contrast unemployment in the community by keeping and reintegrating workers in jobs could also have positive impacts on adverse health outcomes, especially in areas of high crisis intensity.

## Introduction

The ongoing financial and economic turmoil requires policymakers to have a comprehensive understanding of the broad consequences of rising unemployment rates. The medical and health policy literature has raised concerns about the impact of the economic recession on population health, and several recent studies have investigated the effect of the unemployment rate on mortality and healthcare expenditures in Europe after the financial crisis in 2008 [1–4].

The main purpose of this paper is to investigate whether and to what extent the increase in the unemployment rate, due to the recent economic crisis, has impacted hospitalizations and in-hospital mortality associated with acute myocardial infarction (AMI).

Among the different health outcomes, AMI is particularly important in this context. The risk factors for heart attacks are well known and could be affected by the stress and anxiety induced by adverse economic and environmental conditions [5, 6]. In addition, an unfavorable external environment could compound the effect of traditional risk factors, specifically, smoking and obesity [7–10]. In this sense, our study focuses on the short-term impact of the crisis on health, as measured by AMI. The long-term impact of the economic hardships do not represent the target of the present analysis.

Research on the health effects of previous recessions has produced results that seem conflicting. In particular, there is a distinction to be made between individual and aggregated/ecological measures of unemployment.

When individual job loss experience is considered, the literature is consistent in showing that unemployment is associated with poor health, especially when the job-loss is involuntary. For example, the Health and Retirement Survey in the US has been used to show that involuntary job loss negatively and significantly affected both physical functioning and mental health [11] and that it can be considered as a plausible risk factor for cardiovascular and cerebrovascular diseases [12, 13]. In a pioneering study on this issue, Marmot et al provided evidence of a clear inverse relationship between employment grade and cardiovascular mortality [14]. In a prospective cohort study of more than 20,000 employees in Finland, Vahtera et al found that the rate of cardiovascular mortality was 5.1 times higher in the first four years following major downsizing than after no downsizing [15]. A study of banking crises between 1960 and 2002 found a clear association with increased cardiovascular mortality [16].

When studies try to detect a more general (ecological) relation between the economic environment (e.g., unemployment rate) and health, the results are still rather controversial. A recent study of economic fluctuations in Europe, based on aggregate data, showed that worsening employment and other economic indicators (GDP per person, hours worked, and alternative measures of unemployment) affected mortality from specific causes in different ways. A rise in unemployment of 1% was associated with increases in suicides and murders but decreases in road traffic deaths (reflecting lower car use), whereas a rise of 3% or more was associated with an increase in alcohol related deaths and suicides in people younger than 65 years [3, 4]. Several other studies argued that in times of economic hardship and rising unemployment, individuals tend to be more stressed, depressed, and likely to adopt unhealthy lifestyles, such as smoking and unhealthy food consumption, which may increase mortality associated with AMI. In a recent study on 21 Swedish regions, Svensson et al, using panel data estimation, showed that the business cycle effect is insignificant in respect to the overall rates of AMI incidence, mortality and lethality. However, a negative significant effect was found in most model specifications for those of prime working age between 20 and 49 years [6]. Similar results have been found for the economic crisis and smoking behaviors in the USA (8). On the other hand, other ecological studies showed that economic downturns might have the opposite effects on health in high-income countries, i.e., mortality might fall when the economy slows down and might rise when the economy improves [17–19]. This represents the well-known “healthy living in hard times” paradigm, which suggests that in periods of economic crisis, individuals behave better because they are more afraid to lose their jobs and, when unemployed, they take more time for themselves, investing in self-improving and healthy activities. For example, Ruhm showed that one-point fall in unemployment rate is associated with a 1.02%–1.14% increase in AMI deaths [19].

By linking AMI with the unemployment rate at the local level, the present study provides further evidence of the relationship between health and economic shocks as measured at the ecological level.

The study improves the existing evidence in two main directions. First, none of the empirical analyses conducted so far have made use of hospital level administrative data. In the present analysis, AMI cases are identified from individual hospital records from a database with nationwide coverage. Because hospitalization is required for all surviving AMI patients, administrative data are appropriate to capture this phenomenon. Second, much, although not all, of the existing literature has assumed that the economic crisis hits countries uniformly and at the same time [20–22]. This is, however, not realistic. In Italy, it is difficult to identify the exact start date of the crisis in different locations. Moreover, within the country, different areas have been hit with different intensities: while many have not experienced serious increases in unemployment, others have seen dramatic cuts in jobs and employment opportunities. Those studies that have looked at regional differences within countries have yielded important results that would have been missed in national data [20–22]. In order to identify whether sudden worsening of the economic conditions could represent a risk factor for AMI-related conditions, the present analysis exploits the geographic heterogeneity in both the intensity and the timing of the economic crisis. In contrast to the existing literature, no assumptions are made about the strength or the start date of the crisis.

## Methods

### Sources of data and measures

The data were obtained from the hospital discharge database of the Italian Health Ministry.

The current regulations of ethics committees in Italy require only standard written informed consent at the time of hospital admission and anonymous publication of scientific data. Our retrospective observational study fulfilled these requirements and was based on anonymized and de-identified hospital records.

All citizens in Italy are covered by taxed-based public health insurance. Since 1995, hospital care services delivered by public or private accredited hospitals are reimbursed on a “per case” basis, classified according to Diagnosis Related Groups (DRGs). Each group is assigned a specific “value” (tariff) reflecting the intensity of resource consumption needed to treat patients assigned to that group. In order to be reimbursed, a standard discharge record (“Scheda di Dimissione Ospedaliera”) must be completed for each patient. Afterwards, the data are sent to the regional and national Health Authorities for reimbursement. Within this framework, hospitals do not have any incentive to under-report cases. On the other hand, the tight budget constraints in recent years have considerably increased controls and reduced over-reporting.

Discharge records report information on the diagnosis, the treatment, the demographics, the length of stay and the DRG class. Diagnosis and treatment are classified according to the International Classification of Diseases- ninth revision- Clinical Modification (ICD-9-CM) codes. AMI cases were selected by considering ICD-9-CM codes in the range of 410.00–410.92. A total of 942 hospitals, private clinics and nursing homes have been reimbursed for AMI at least once in the observation period. In order to focus only on the most reliable information, institutions with less than 10 AMI cases per year were omitted from the sample (equivalent to 0.9% of the total number of hospitalizations at the national level). A total of 549 hospitals were selected for the analysis. Each hospital was observed on a yearly basis from 2009 until 2012, with the exception of 30 hospitals for which one year of observation was missing. It is important here to note that these hospitals did not close during the observation period. Four dependent variables are available for patients with a diagnosis of AMI: the number of cases per hospital, the number of hospital days, the in-hospital mortality and the expenditure evaluated by the DRG tariffs. The number of AMI cases and the in-hospital mortality are also available by sex and age groups.

Each hospital is geographically located within a “Sistema Locale del Lavoro” (SLL). A SLL is defined by the Italian National Statistical Office (ISTAT) and aggregates all the neighboring “comuni” (towns) with a common economic structure. As defined, a SLL is thus the ideal geographical unit of analysis for the purpose of this study. There are 686 SLLs across Italy, with an average of 11.7 towns in each SLL. As is standard practice, hospitals are located in the most populated areas, so that only 319 SLLs are actually included in the analysis. For each SLL, increases in the annual unemployment rates provided by ISTAT were used as a measure of the area-specific crisis intensity. Note that by considering the intensity of the changes in unemployment in a five-year period, the analysis did not rely on a priori definition of crisis. In particular, no assumption was made about the exact timing in which the crisis had hit the country or a specific area.

### Study design

The areas (SLLs) were categorized into quintiles according to the changes in their unemployment rates (i.e., crisis intensity). The first quintile included all of the SLLs associated with a very low crisis intensity, with a mean increase in unemployment of 0.6% (for the second quintile, the average increase in the unemployment rate is 1.8%). The fifth quintile, in contrast, included the areas strongly hit by the economic crisis, with a relevant average increase in the unemployment rate of 7.2% (3.6% for the fourth quintile).

The analysis uses standard difference-in-differences methodology. It sets the two lowest quintiles as the control group and the highest two quintiles as the case group. It then compares the changes in the outcome variables (hospitalizations, hospital days, in-hospital mortality, the length of stay, and expenditures) across the different quintiles. This approach is used both for the rough data and for the predictions, where data are adjusted for a number of control variables.

Considering changes rather than levels provides two main advantages. First, the analysis is focused on the within hospital variation only. Hence, time-invariant, hospital specific structural characteristics, even if unobserved, should not affect the results. Second, it is clear that SLLs and hospitals might differ substantially in their AMI prevalence and incidence. The approach taken here controls for this geographic heterogeneity in the levels of AMI by excluding this source of variation.

### Data analysis

Simple means of the differences-in-differences analysis provide reliable results. Additional covariates, however, are added to the analysis in order to better control for time-changing and non-hospital-specific factors that might affect the results. Non-linear secular trends are crucial and are considered by introducing three year-dummies. In addition, province fixed effects are used in order to control for institutional characteristics that might affect how both the economic crisis and AMIs are managed. The logs of the area-specific population sizes were included in order to control for changes in the size of the population, although it should be noted that mobility is rather low among Italians and no substantial migration from economically depressed areas was observed. When possible (i.e., when non-linear models are used), the type of hospital (private vs public) was also included in the analysis.

Predictions were calculated by running panel-data models including the abovementioned covariates together with dummies of the quintiles of crisis intensity and the interaction between these and the year-dummies. The precise specification of the model is reported in S1 Appendix. Because three out of four of the outcome variables, namely hospital days, hospitalizations, and in-hospital mortality, are positive integer numbers, Poisson panel data models were considered. For expenditures and length-of-stay, linear fixed effect panel data models were used. Once the models were run, predictions were performed and differences in the changes across quintiles of crisis intensity were reported and statistically tested.

## Results

Descriptive statistics for the available data are reported in Table 1. The information is divided into two periods, and the differences are tested. On average, each hospital registered 223 AMI cases per year, with more than 1700 hospital days, and a mean length of stay of approximately 8 days per AMI admission. The average in-hospital mortality was approximately 6.5%. When evaluated according to the DRG tariffs, these figures were associated with an average expenditure to the regional health systems of more than 1.4 million euros per hospital per year.

**Table 1:** Acute Myocardial Infarction in Italy: descriptive statistics and trends of the main variables ^a^.

	Mean for2009 & 2010	Mean for2011 & 2012	P-value^b^
**Mean N. hospitalizations**	222.95	216.68	0.55
**Mean N. hospital days**	1745.92	1683.99	0.37
**Mean length of stay (days)**	8.25	8.15	0.51
**N of In-hosp-deaths**	13.86	13.71	0.82
**In-Hospital deaths %**	6.35	6.69	0.18
**Expenditure(million euros)**	1.39	1.58	0.02
**Average cost of hospitalization(euros)**	5464.58	5942.14	0.000
***Hospitalizations***			
**Under 65 years**	77.22	74.58	0.42
**Over 65 years**	145.73	142.10	0.50
**Females**	76.56	75.46	0.69
**Males**	146.39	131.61	0.36
***In-hospital deaths***			
**Under 65 years**	1.18	1.13	0.60
**Over 65 years**	12.53	12.72	0.76
**Females**	6.72	6.81	0.78
**Males**	6.99	7.04	0.87

^a^ Values are per-hospital per-year.

^b^ T-test for mean differences.

Table 1 shows the absence of a significant increase in AMI occurrence due to the economic crisis. Interestingly, the number of hospitalizations and the number of hospital days did not change over time. Mortality increased slightly, although the change was not statistically significant. Data on hospitalizations and in-hospital mortality are available for sex and age subgroups. Their distribution, as expected, reflected the higher incidence and mortality for males and for patients aged more than 65 years. Finally, changes in the main variables within the gender and age subgroups were never significantly different at the national level.

Although descriptive statistics did not reveal an overall significant increase in AMI occurrence between 2009 and 2012, when geographic variations and the comparison across areas with a different unemployment rate were considered, the impact of the economic crisis on AMI episodes is more reliably described.

**Fig 1:**
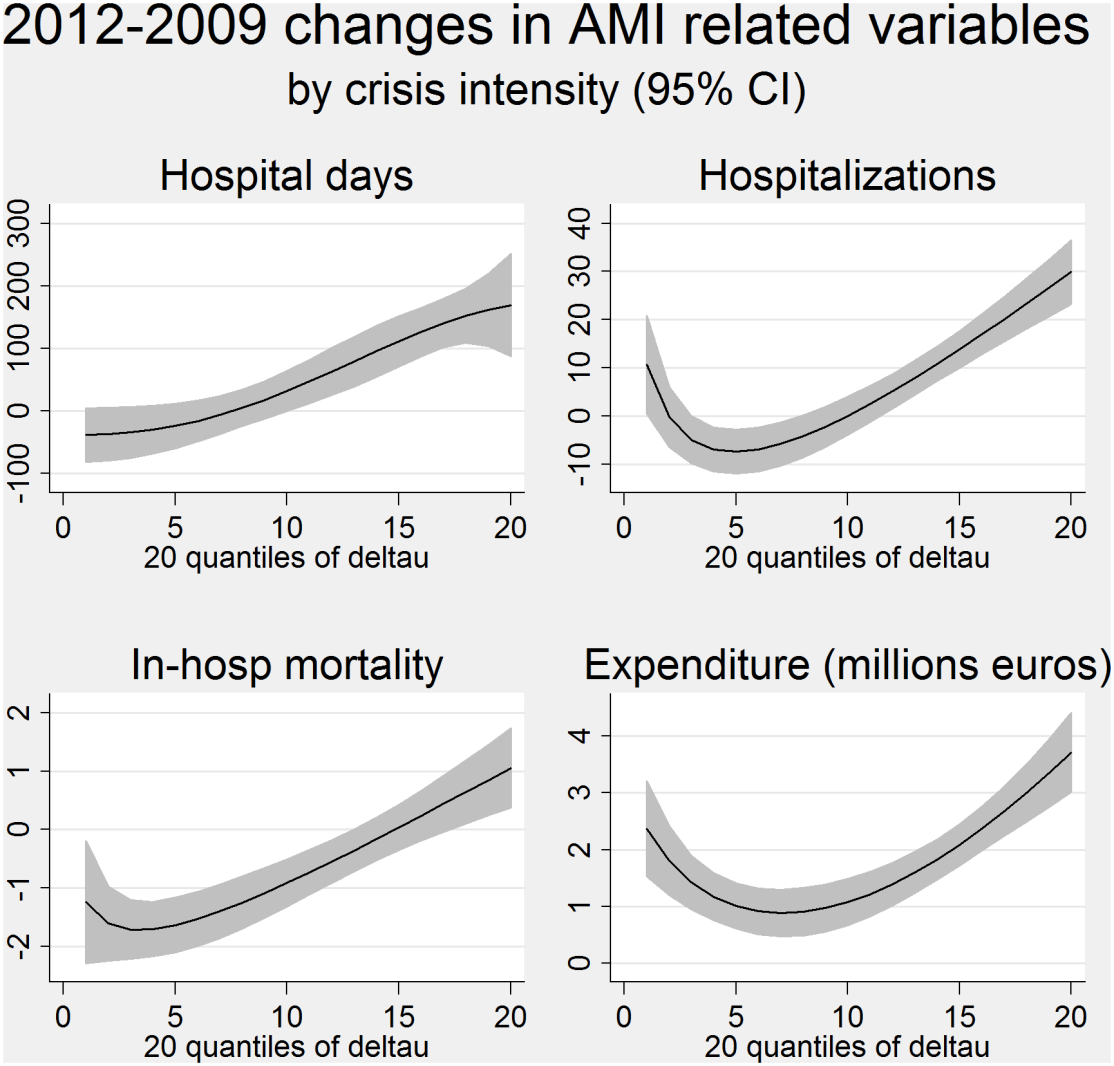
The fractional polynomial fit of changes in the outcome quintiles (20) of crisis intensity, with 95% confidence intervals.

Fig 1 shows the relationship between the increase in the four outcome measures and the increase in area-specific unemployment rates (i.e., the crisis intensity). The graph is based on the fractional polynomial fit, with 95% confidence intervals. The relationship between AMIs and crisis intensity is clearly not linear: it decreases for low levels of crisis intensity and increases in the highest level of unemployment rate. This relationship was evident for all the outcomes except for the length of hospital stay.

**Fig 2:**
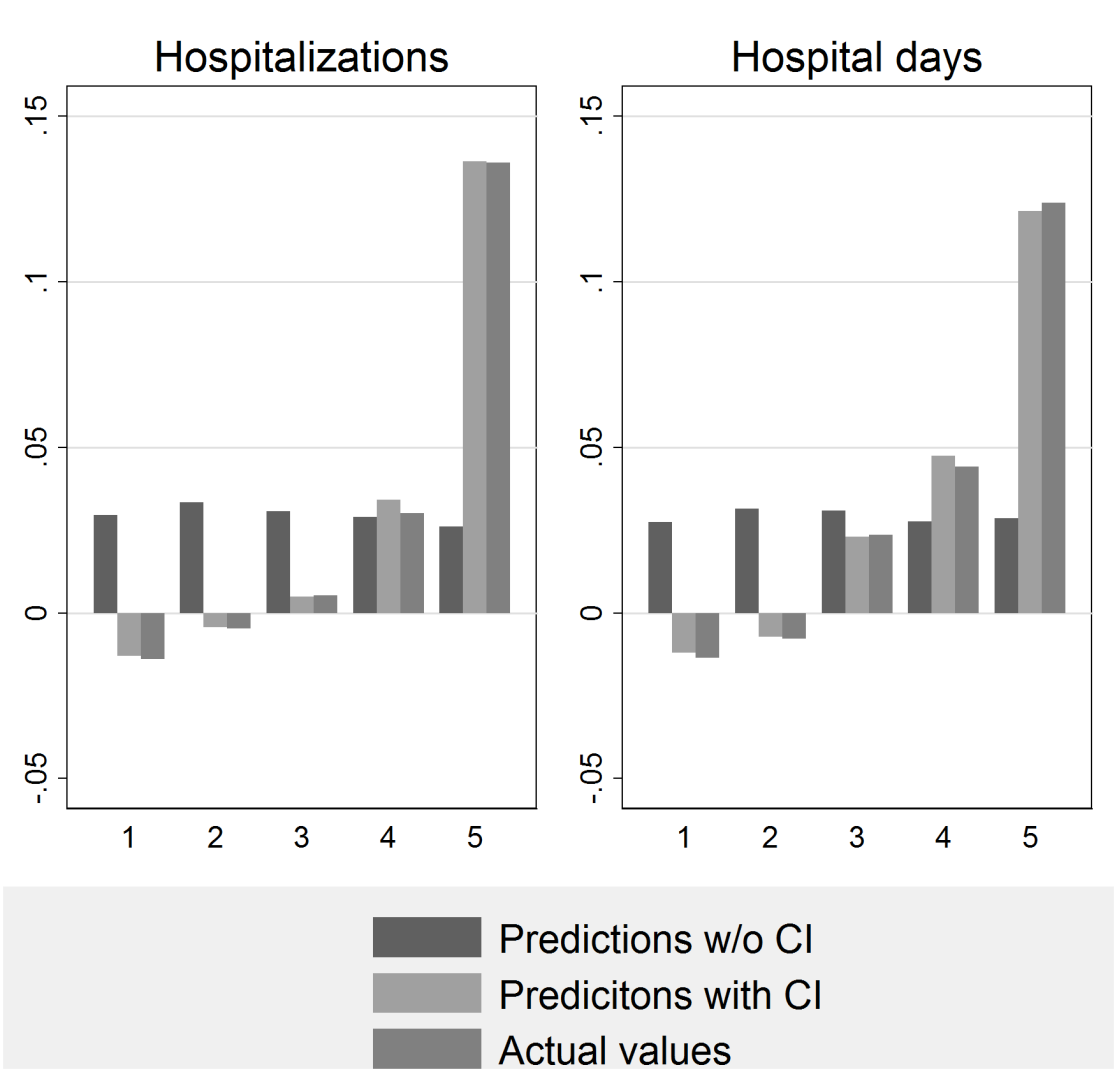
The actual and predicted % changes in hospitalizations and hospital days, by quintiles of crisis intensity. Predictions without Crisis Intensity (CI) do not include dummies for the quintiles of CI.

In Fig 2, the actual and predicted increases in AMI occurrence are reported by quintiles of crisis intensity. Fig 2 shows that, when predictions do not take into account the strength of the economic crisis (i.e., predictions without crisis intensity), they under-estimate the increase in AMIs in the top quintiles areas.

Table 2 provides the results from the difference-in-differences analysis. It compares both the predicted and actual values across case and control groups. Because the first two quintiles are associated with lower increases in AMI occurrence (see Figs 1 and 2), these areas were used as the “control group” against which to test the changes in the outcome variables in the areas more exposed to the economic crisis. Two case groups are used. We report both the absolute and percentage change values between 2009 and 2012. The results of the differences in the mean differences (t-test) analyses are reported in the p-value columns.

**Table 2:** The difference-in-differences analysis of the predicted and actual (in parenthesis) values^a^.

	Control Group		Case Group 1		Case-Control diff.	Case Group 2		Case-Control diff.
	1^st^ + 2^nd^ quintiles of CI		5^th^ quintile of CI			4^th^ quintile of CI		
	Mean Increase2012-2009	% Increase2012-2009	Mean Increase2012-2009	% Increase2012-2009	**Mean increase (P-value** ^b^ **)**	Mean Increase2012-2009	% Increase2012-2009	**Mean increase (P-value** ^b^ **)**
**Days in hospital**	-20.47(-20.73)	-0.09(-1.09)	179.31(197.54)	12.14(12.38)	<0.001(<0.001)	68.58(67.00)	4.74(4.41)	<0.001(0.092)
**Length of Stay**	0.09 (0.09)	1.09 (1.10)	0.38(0.38)	5.64(5.67)	<0.001(0.391)	0.26(0.25)	3.00(3.01)	<0.001(0.181)
**Expenditure(million euros)**	0.16(0.16)	10.4(10.1)	0.52(0.52)	46.6(46.5)	<0.001(<0.001)	0.27(0.26)	15.51(15.04)	<0.001(0.317)
***Hospitalizations***								
**Total population**	-2.17(-2.28)	-0.09(-0.09)	28.65(28.94)	13.62(13.63)	<0.001(<0.001)	6.75(6.23)	3.40 (3.01)	<0.001(0.133)
**Under 65 years**	-0.83(-0.90)	-1.03(-1.14)	12.22 (12.38)	14.39 (14.61)	<0.001 (<0.001)	4.10 (3.65)	6.06 (5.24)	<0.001 (0.089)
**Over 65 years**	-1.26(-1.37)	-0.08(-0.08)	16.60(16.56)	13.27(13.16)	<0.001(<0.001)	2.90(2.57)	2.23(1.87)	<0.001(0.215)
**Females**	-1.47(-1.47)	-1.73(-1.7)	7.32 (7.25)	10.08(10.55)	<0.001(<0.001)	1.06(0.58)	1.52 (0.07)	<0.001(0.229)
**Males**	-0.68(-0.80)	-0.04(-0.05)	21.33(21.39)	15.12(15.05)	<0.001(<0.001)	5.80(5.64)	4.52(4.21)	<0.001(0.115)
***In-hospital mortality***								
**Total population**	-1.60(-0.46)	-9.78(-9.78)	0.85(0.82)	8.70(7.90)	<0.001(<0.001)	-0.01 (-0.10)	0.00(0.50)	<0.001 (0.051)
**Under 65 years**	-0.17(-0.18)	-18.09(-13.52)	0.06 (0.07)	8.81 (6.01)	<0.001 (0.001)	0.25 (0.25)	50.00 (26.77)	<0.001 (0.016)
**Over 65 years**	-1.43(-1.37)	-10.37(-9.34)	0.79(0.75)	10.03(8.29)	<0.001(0.001)	-0.28(-0.34)	-2.54(-2.82)	<0.001(0.112)
**Females**	-0.87(-0.88)	-11.23(-8.5)	0.43(0.50)	10.23(10.91)	<0.001(0.002)	-0.07(-0.09)	-1.21(-0.00)	<0.001(0.060)
**Males**	-0.57(-0.69)	-7.52(-11.01)	0.44(0.32)	8.70(5.43)	<0.001(0.003)	0.00(-0.01)	0.01(-0.00)	<0.001(0.121)

^a^ The values represent the predictions per hospital per year based on panel-data regressions. Regression for “expenditure” is a linear panel data fixed effect. The others are Poisson random effects. All regressions include 4 quintiles dummies, 3 year-dummies, the interaction between quintiles and years, province-level dummies, the log of the population per year and a private hospital indicator. The actual values from the original data are presented in parentheses.

^b^ The p-value for the t-test on the differences between the mean increase in the case group and the mean increase in the control group.

As an example of how to read the results from Table 2, consider the first outcome, “Days in hospital”. From the table, we read that, between 2009 and 2012, the per-hospital number of AMI days in the control group (areas less affected by the crisis) decreased on average by 20 units (or 0.9%), while in the first case group (5^th^ quintile), it increased by a striking 179 units (or 12%). The P-value column presents the difference in these average changes between the case and the control group and confirms that, obviously, this difference is strongly significant. When another, milder, case group is considered (the 4^th^ quintile), the results are weaker but still significant.

The actual and predicted values are remarkably similar, especially for the 5^th^ quintile, and show the strong differential impact of the crisis on the areas more strongly exposed to the increased unemployment rates. Hospitals in the areas within the fifth quintile of crisis intensity had an increase of approximately 30 AMI cases (approximately 13%) with respect to hospitals in all other quintiles (p<0.001). Notably, this difference is entirely due to the increase in AMI in the case areas: on average, hospitals in areas not hit by the crisis did not register any significant increase in the number of AMI cases. The same is true for the number of hospital days (an increase of almost 200 days, p<0.001). Because the average length of stay increased only slightly (and not significantly in the rough data), the increase in the number of hospital days reflects the increase in the number of hospitalizations. In-hospital mortality in the bottom quintiles was reduced by almost 10% between 2009 and 2012, while the increase in highly affected areas (the 5^th^ quintile) was approximately 9% (an increase of 2.4 in-hospital deaths per hospital per year, p<0.001). Such a marked variation reflects the low numbers involved in these outcomes, but the trend in mortality reduction in the control group hospitals was, in this case, particularly evident. When the 4^th^ quintile is considered as the case group, the differences were significant but smaller in magnitude.

The increase in the incidence of AMIs was translated into an increase of around €350.000 (p<0.001) in expenditures per hospital per year. When the analysis was extended to sex and age subgroups, all the differences were always statistically significant. Importantly, the groups that suffered most in terms of increases in AMI hospitalizations were males between 44 and 65 years old, who were the most directly exposed to the economic and social effects of the increasing rate of unemployment. In-hospital mortality increased more for women than for men, although only slightly.

## Discussion

The aim of the present analysis was to investigate the relation between the unemployment rate and the incidence of hospital level AMI in the Italian NHS. The results show that there is a clear and strong pattern: hospitalizations and expenditures between 2009 and 2012 increased significantly in the areas where the crisis hit more strongly. Overall, compared with the other hospitals in the country, in the 107 hospitals situated in the crisis-exposed areas (the fifth quintile), 30 additional (13%) AMI cases, 200 additional hospital days (13%) and 2 additional in-hospital deaths (17%) were observed. This finding translated into an increase in the financial pressure on the system: each hospital in the areas most exposed to the crisis spent an average of 350,000 euros more for AMI hospitalizations.

The impact of the crisis on the 4^th^ quintile is weaker. Moving down the “crisis intensity” scale, the role of the economic environment tends to lose importance in predicting changes in AMI occurrence. Accordingly, when the 3^rd^ quintile was considered, no further significant difference was detected (results not shown).

These results provide a more precise estimation of the impact of the crisis on the burden of AMI in areas heavily affected by an increasing unemployment rate. In 2012, the crisis was associated with approximately 4,100 additional AMI hospitalizations in the 215 hospitals located in the 5^th^ and the 4^th^ quintile areas of crisis intensity. This figure corresponds to an 8.2% increase in the 2009 per-hospital average number of AMIs. In-hospital mortality increased even more. Compared to what might be expected without the crisis (i.e., a reduction in mortality), hospitals in the two top quintiles experienced an estimated total of 400 additional deaths (14% more with respect to 2009). Further, the 215 hospitals in the top quintiles spent approximately 30% more on treating AMI patients than the hospitals in the lowest quintiles, with a total of approximately 100 million additional euros spent at the national level.

The results are not trivial because they capture the impact of the economic environment on individual health, clearly showing that economic-related issues are sources of public health concern, even in the short-term. Indeed, the present study focuses on the impact of the crisis on health in the two years following the beginning of a strong economic crisis. The long-term impact of the economic hardships, channeled mainly through riskier health behaviors and the chronicity of stress, are certainly important but do not represent the target of the present analysis.

The stress induced by overall economic uncertainty and sudden job loss is probably the main transmission mechanism from the economic environment to individual health. Notably, the stress from economic hardship does not necessarily directly affect the person who lost her/his job. From one perspective, a high level of unemployment increases job insecurity, so that one can feel threatened even without directly experiencing unemployment. From another perspective, insecurity affects the entire household. In Italy, unemployment benefits exist only for a portion of employers and are time-limited. Typically, families rely on the wealth accumulated by the older generations. Women and men over 65 years of age, although not directly affected by the crisis, might thus suffer as well from these stressful conditions.

Note also that the results provided here focus on the average relations between the economic environment and health. According to the existing consensus statement (e.g., Marmot and Wilkinson, Social Determinant of Health, Oxford University Press, 2005), socio-economic characteristics such as education, wealth and social class can, and probably do, play an important role in determining the health of a person, and they are also likely to influence the relationship between an economic shock and most health indicators. Identifying the main socioeconomic protectors in the presence of an economic crisis can thus represent an interesting topic for further research, but it goes beyond the objectives of the present analysis.

Our results reinforce the body of evidence in the literature that suggests “this time is different”, by showing that the present and ongoing crisis has an impact beyond well-known variables such as suicides [4, 23, 24], HIV [25] or health behaviors and preventive activities [26, 27]. It is indeed becoming clear that the present economic hardships might produce important hidden individual and social consequences that are likely to be underestimated by official economic statistics. Efforts in the public health and economics literature should thus move more towards a better estimation of these human and economic costs.

Although in line with the most recent public health reports, these results might seem at odds with the standard paradigm of “healthy living in hard times” dominating in the social science and economics literature. In addition, our findings also provide evidence that the relationship between the crisis intensity and occurrence and the impact on AMI incidence is U-shaped. Hospitalizations for AMI remain stable (or decrease) in areas less affected by the crisis. Because the economic shock is not particularly strong, the uncertainty regarding present and future jobs is not heavily affected. In these areas, the incidence of AMI does not increase (and can even reduce), as predicted by the “healthy living in hard times” literature. In contrast, unemployment rates suddenly “explode” where the crisis hits hard, a scenario that might be very hard to address for many reasons. In these areas, the entire economic structure is threatened, and job uncertainty increases to levels never experienced before. The burden of stress-related AMI incidence increases as well, as predicted by the “this time is different” literature. This non-linear relationship between economic and health shocks can thus explain much of the apparent inconsistencies between different streams of literature, as already noted by a very recent study focusing on mortality in Europe [28].

From a health policy perspective, our results show that the economic crisis puts hospitals under a strong pressure, stemming from both the reduction in expenditures and increases in demand. This is happening at a time when the budgets for both welfare and healthcare interventions are being cut in the majority of southern European countries. Under these circumstances, it is particularly difficult to see budget cuts as useful ways of increasing the efficiency of the system. The evidence provided in our analysis can be of value to politicians and policy-makers who should be well-informed about the actual consequences of their public finance decisions. While designing policies to cut healthcare expenditures in times of crisis, policy makers should be aware of the potential impact on the health of communities. Policies aimed to contrast unemployment in the community by keeping and reintegrating workers in jobs could also have positive impacts on adverse health outcomes associated with the economic crisis, especially in areas with high crisis intensity.

The present analysis has four main limitations. First, the data are not based on the area of residence of the patient. It is thus not possible to identify the precise number of people served by each hospital and to quantify the relative risk associated with living in areas heavily affected by the crisis. In particular, our results would be upward biased if people in low-crisis areas moved to hospitals in high-crisis areas, something that is highly unlikely to have happened on a systematic level. In more detail, a problem would arise if patients chose hospitals according to the quality and the best hospitals are located in the areas highly affected by the crisis. In this case, the results could just signal an increase in AMI incidence for good quality hospitals (e.g., teaching hospitals). Although one cannot exclude this possibility, it should also be considered that AMI is not an elective procedure. Patients are thus normally allocated to hospitals depending mainly on the time needed to access the treatment, which is approximated by distance. In addition, there is no evidence that better hospitals are located in areas highly affected by the crisis. Rather, they are usually found in cities, which are, on average, less affected by the crisis, especially when compared to southern, less urbanized SLLs. In conclusion, although the analysis would benefit from a more precise geo-localization of the AMI cases, we believe that it nevertheless provides a rather reasonable approximation of reality. The strong significance of the results indicates that unemployment rates at the SLL level do capture something relevant for the description of AMI patterns.

A second limitation is the observational nature of the data. Different hospitals and practitioners might classify cases in different ways. Although this is unavoidable, our analysis implemented different approaches to limit this problem. The AMI cases were aggregated at a broad level, so that small differences in the interpretation of the ICD coding and in the compilation of the discharge records should not have influenced the outcome variables. Additionally, panel data techniques were used, and the data were controlled at the hospital level. Importantly, we measured the variations of AMI cases within hospitals. Unless procedures and practitioners inside each hospital varied between 2009 and 2012, the changes in outcomes at the hospital level should not be strongly affected by this problem. One further limitation arises from the data used: hospital records do not represent the totality of AMI mortality because people can die before reaching the hospital. However, the financial pressure on the system results mainly from the treatment of patients with AMI who are admitted to the hospital. In addition, we didn’t have 2012 data for 30 hospitals of our sample (approximately 1% of total number of observations). Last but not least, this study suffers from the same limitation that affects the related literature: an association does not necessarily imply causation. It is worth noting, however, that the economic crisis is entirely exogenous with respect to individual decisions. The case described here can thus be seen as a “natural experiment”, something that reduces possible endogeneity biases.

In conclusion, more attention should be given to the increase in health needs associated with the financial crisis and to the consequent additional financial pressure imposed on health systems.

## Supporting Information

S1 Appendix (DOCX)
