# Supplementary material for: The Economic Crisis and Acute Myocardial Infarction: New Evidence Using Hospital-Level Data
Source: PLoS One. 2015 Nov 17;10(11):e0142810. doi: 10.1371/journal.pone.0142810 (PMC4648494; doi:10.1371/journal.pone.0142810)
Supplement: S1 Appendix — (DOCX) [file pone.0142810.s001.docx]

**Appendix**

The empirical model used to develop predictions is the following:

$$y_{ijt}=\alpha+{\delta_{j}+\gamma_{t}+{qci}_{i}+\left( {qci}_{i}*\gamma_{t} \right)+\beta*log(pop)}_{it}+{type}_{i}+\varepsilon_{ijt}$$

where y is the outcome of interest for hospital *i* in province *j* in year *t*; δ are province level fixed-effects (102 dummies for 102 provinces), γ are year dummies (2010,2011,2012), *qci* is the quintile of crisis intensity of the SLL of the hospital, *log(pop)* is the population of the SLL of the hospital in year *t*, *type* can be either private or public and ε is the error term. The saturated model thus includes a total of more than 120 variables. The short version of the model used in Figured 2 does not include the qci variables (or their interaction with the year dummies).

Estimation for count variables (hospitalizations, hospital days and mortality) is through Poisson panel-data random effects. With costs and length of stay, the outcome is analysed through a linear panel data model, where the fixed-effect approach (where, formally, α is replaced by $\alpha_{i})$can “demean” the variables and get rid of all the time-invariant variation without including further dummies.

Once the model is run, absolute predicted changes in outcome variables, for each hospital *i* in province *j*, are calculated as $E\left( y_{2012} \right)-E\left( y_{2009} \right)$, while the percentage changes are $\frac{E\left( y_{2012} \right)-E(y_{2009})}{E(y_{2009)}}$. Statistical tests over the differences in changes in different quintiles areas are performed as regular t-tests.
